# Astaxanthin exerts neuroprotection against experimental intracerebral hemorrhage in mice: association with anti-neuroinflammation and the Nrf2-ARE pathway

**DOI:** 10.3389/fneur.2026.1879830

**Published:** 2026-07-31

**Authors:** Xingfen Su, Lijin Wu, Fuxiang Chen

**Affiliations:** 1 Department of Neurosurgery, The First Affiliated Hospital of Fujian Medical University, Fujian Medical University, Fuzhou, Fujian, China; 2 Department of Neurosurgery, National Regional Medical Center, Binhai Campus of the First Affiliated Hospital, Fujian Medical University, Fuzhou, China

**Keywords:** anti-neuroinflammation, astaxanthin, intraceryebral hemorrhage, neuroprotection, Nrf2-ARE pathway

## Abstract

Astaxanthin (ATX) is a dietary carotenoid that possesses potent effects of anti-oxidation, anti-inflammation, and anti-cell death. ATX has neuroprotective properties in a variety of neurological diseases. However, whether ATX has a neuroprotective effect in intracerebral hemorrhage (ICH) is still unknown. The present study was carried out to explore the protective effects of ATX and the underlying mechanisms using a collagenase-induced ICH in mice. Single dose of astaxanthin (100 mg/kg) or vehicle was administrated to mice through intraperitoneal injection at 30 min after ICH induction. Our results showed that ATX significantly ameliorated secondary brain injury after ICH, including neurological deficits, neural cell degeneration, blood–brain barrier (BBB) disruption and brain water content. We further demonstrated that ATX exerted anti-neuroinflammatory effects by inhibiting microglia activation and production of pro-inflammatory mediators (TNF-α and IL-1β). ATX administration attenuated ICH-induced oxidative stress by reducing the level of malondialdehyde (MDA) and restoring the activities of glutathione peroxidase (GPx) and superoxide dismutase (SOD). Moreover, ATX promoted nuclear factor erythroid 2-related factor 2 (Nrf2) protein translocation from cytoplasm to nucleus and increased the expression of Nrf2-antioxidant response element (ARE) downstream factors such as heme oxygenase-1 (HO-1) and NADPH quinine oxidoreductase-1 (NQO-1) after ICH. Taken together, these data provided the first evidence that astaxanthin exerted neuroprotective effects in ICH mice, which suggests the Nrf2-ARE pathway was significantly correlated with the neuroprotective effects of ATX.

## 1 Introduction

Intracerebral hemorrhage (ICH) accounts for 20–30% of stroke patients in Asia (1). ICH is associated with high rates of morbidity and mortality and considered as the most severe form of stroke (1). Despite intensive research, no effective medical treatment is currently available for ICH patients (2). Therefore, it is of critical importance to identify novel therapeutic targets and develop new medical agents for ICH patients.

Astaxanthin (ATX), known as a strong antioxidant, has a variety of pharmacological properties, including anti-oxidative stress, anti-inflammation, and anti-cell death (3). ATX has been reported to exert neuroprotection in several central nervous system (CNS) diseases, including traumatic brain injury (TBI), ischemic stroke and subarachnoid hemorrhage (SAH) (3–5). However, accumulating preclinical studies have demonstrated that astaxanthin exerts potent neuroprotective effects in multiple neurological and neuropsychiatric disorders, including Alzheimer’s disease, Parkinson’s disease, neuropathic pain and autism, mainly via suppressing neuroinflammation, alleviating oxidative stress and regulating mitochondrial function (6). A series of pharmacological studies further elaborated the multi-target mechanisms of astaxanthin in central nervous system diseases, covering NF-κB, MAPK and Nrf2-ARE signaling cascades (7). Recent translational researches on cerebrovascular diseases also explored the optimal dosage and administration regimens of astaxanthin for stroke and related brain injury (8, 9). Despite the above advances, whether ATX has a neuroprotective effect in intracerebral hemorrhage (ICH) and its exact underlying mechanism remains poorly understood.

Nuclear factor erythroid 2-related factor 2 (Nrf2)- antioxidant response element (ARE) pathway, as a key player in antioxidative homeostasis, has been proven to exert neuroprotection in ICH (10, 11). Nrf2 further induces the expression of many downstream factors, such as heme oxygenase-1 (HO-1) and NADPH:quinine oxidoreductase-1 (NQO-1) (12). A recent study showed that ATX attenuated early brain injury through Nrf2-ARE pathway after SAH in rats (13). However, it is still unknown whether Nrf2-ARE pathway is involved in the protective mechanism of ATX after ICH. Therefore, the purpose of the present study was to investigate the neuroprotective effects of ATX in reducing ICH-induced brain injury in mice and explored the possible involvement of Nrf2-ARE pathway in the putative neuroprotection of ATX.

## 2 Materials and methods

### 2.1 Animals and ICH model

All experiments used male C57BL/6 mice. Accumulated evidence has demonstrated that sex differences exert profound impacts on the pathophysiology of intracerebral hemorrhage, which may affect the generalization of our experimental results. All the animal experiment procedures were carried out in compliance with the guidelines of National Institutes of Health and were approved by the Animal Care and Ethics Committee of Fujian Medical University. Male C57BL/6 mice (weighing 28–32 g) were purchased from the SLAC Laboratory Animal Co., Ltd. (Shanghai, China). Animals were housed in a light- and temperature-controlled environment, and were allowed free access to water and food. All mice were randomly divided into three experimental groups using a computer-generated random number table. All surgical operations, sample testing and data analysis were performed by researchers blinded to group allocation to eliminate observer bias. Sample size was calculated based on prior *in vivo* neuropharmacology studies for brain injury models. A total of 6 mice were assigned to each group (n = 6 per group), which was sufficient to detect biological differences in neurological function, histopathology and molecular indicators with conventional statistical power.

The mice ICH was induced using a collagenase injection model as our previous study (14). Briefly, mice were anaesthetized with isoflurane (4% for induction and 2% for maintenance) and fixed prone in a stereotactic frame. A 1 mm cranial burr hole was drilled at 2 mm right lateral to the bregma, and a 2-μl microsyringe was inserted 3.5 mm below the dura. A total of 0.06 U of bacterial collagenase IV (Sigma-Aldrich, St. Louis, MO, USA) which dissolved in 0.5 μL saline was infused into the right basal ganglia (coordinates: 2 mm lateral to the bregma, 3.5 mm in depth). The needle was left for an additional 5 min and withdrawn slowly to prevent reflux. Sham-operated animals were injected with an equivalent volume of saline solution instead of collagenase. Rectal temperature was maintained at 37 °C with a heating pad throughout surgery. Animals were allowed to full recover under observation. Brain tissues of perihematomal regions were collected for further analysis.

### 2.2 Experimental design and astaxanthin administration

**Figure 1:**
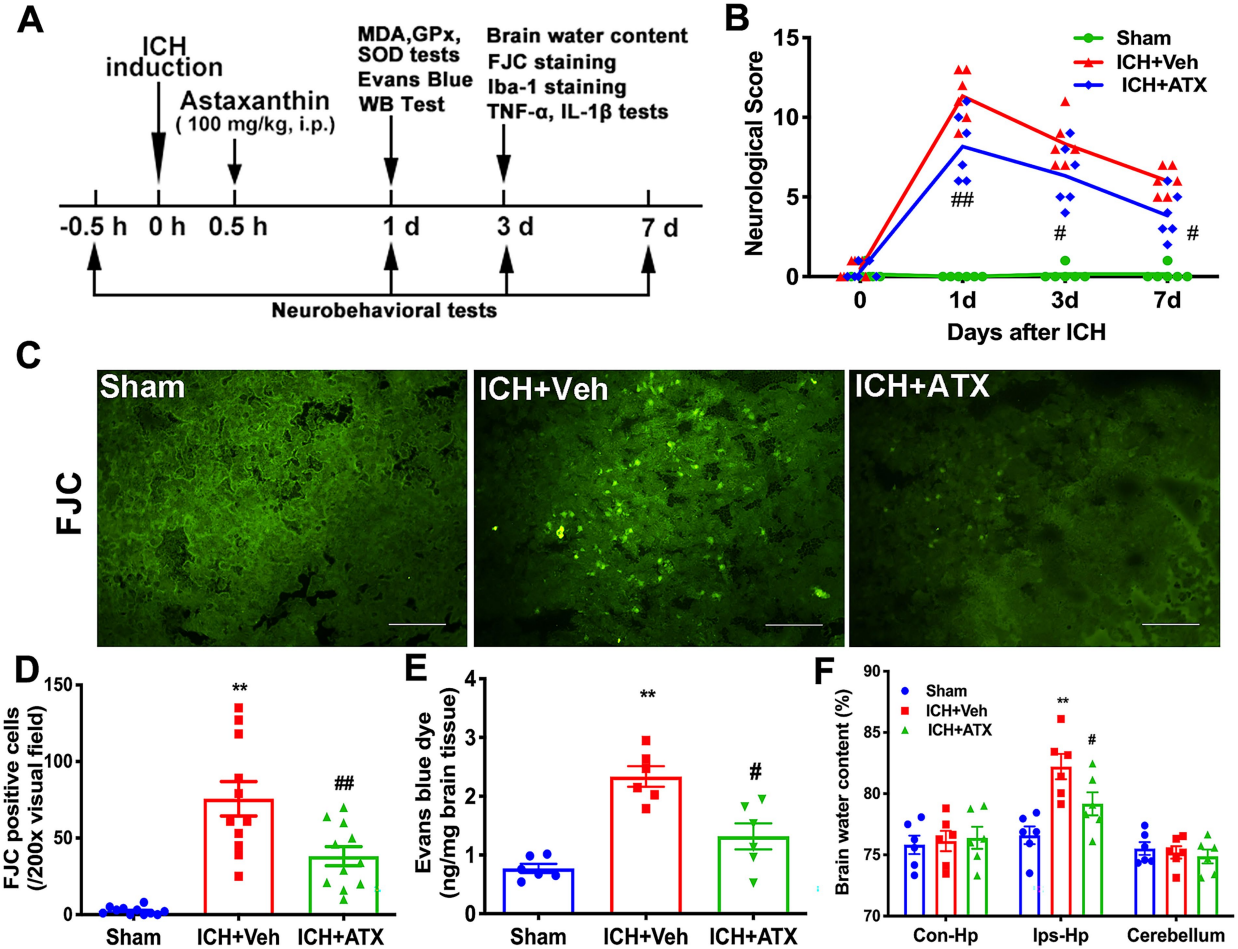
Administration of astaxanthin (ATX) protected mice against brain injury after ICH **(A)** The experiment workflow. **(B)** Statistical analysis showed that ATX treatment significantly reduced neurological deficits at all assessed time points after ICH. **(C)** Representative pictures of Fluoro-Jade C (FJC) staining (green) showed neural cell degeneration at 3 days after ICH. Scale bar, 50 μm. **(D)** Quantification of FJC-positive cells showed that treatment with ATX significantly reduced the neural cell degeneration at 3 days after ICH. **(E)** The blood–brain barrier disruption was assessed by Evans blue (EB) dye extravasation at 24 h post ICH. **(F)** Brain edema was evaluated through measuring brain water content at 3 days after ICH. Con-Hp, contralateral hemisphere; Ips-Hp, ipsilateral hemisphere. Data were presented as mean ± SEM. Individual data points of each mouse are overlaid on the plots to reflect data variability and sample distribution. *n* = 6 mice/group. **p < 0.01 versus Sham group; ^#^*p* < 0.05, ^##^*p* < 0.01 versus ICH + Veh group.

A schematic of experimental procedures was shown in Figure 1A. Mice were randomly assigned to three groups: Sham operated group (Sham), ICH treated with vehicle group (ICH + Veh), and ICH treated with astaxanthin group (ICH + ATX).

Astaxanthin (Santa Cruz Biotechnology, USA, 98% purity) was dissolved in corn oil (1 mL/kg) immediately before use. To test the neuroprotection of ATX in ICH, astaxanthin (100 mg/kg body weight) was administered through intraperitoneal injection 30 min after ICH modeling. In the present study, a single 100 mg/kg dose was selected with reference to multiple published preclinical studies on brain trauma, subarachnoid hemorrhage and cerebral ischemia, where this dosage exerted stable neuroprotective effects without obvious toxic reactions in mice (3, 5). The 30 min post-ICH administration time was chosen because the early stage of hemorrhage is the critical window for the initiation of secondary brain injury, including oxidative stress and neuroinflammation. The other two groups were treated with an equal volume of corn oil 30 min after surgery.

In this study, different detection time points were set according to the pathological characteristics of ICH: Neurological function, Evans blue (EB) extravasation and oxidative stress indicators were detected at 24 h post-ICH (early secondary injury stage); Brain water content, microglial activation and inflammatory cytokines were detected at 3 days post-ICH (peak period of brain edema and neuroinflammation); Neural cell degeneration was assessed at 3 days post-ICH.

### 2.3 Neurobehavioral tests

A 24-point neurological scoring system was used to evaluate the functional outcomes in mice after ICH. This system contains six individual tests including body symmetry, gait, climbing, circling behavior, front limb symmetry and compulsory circling as our previous reports (14, 15). Mice were graded from 0 to 4 points with increasing severity in each test by an observer blinded to the treatment groups. The neurobehavioral function was tested at half hour before surgery (0 day) and at 1 day, 3 days, and 7 days after ICH.

### 2.4 Fluoro-Jade C staining

Fluoro-Jade C (FJC, a marker for degenerating neurons) labeling was performed using a protocol as it was previously reported (15). In brief, frozen sections were prepared, fixed and mounted on slides. Slides were first immersed in 1% sodium hydroxide in 80% ethanol for 5 min. Then slides were rinsed with 70% ethanol for 2 min, in distilled water for 2 min, followed by incubating in 0.06% potassium permanganate solution for 10 min. After rinsing with distilled water for 2 min, slides were incubated in 0.0001% FJC solution (Chemicon, USA) for 10 min. Slides were rinsed with distilled water three times, air-dried, coverslipped and visualized by two pathologists who blinded to the experimental groups under a fluorescence microscope (Eclipse Ti, Nikon Corporation, Tokyo, Japan). The data were expressed as FJC-positive cells/(200 magnification visual field).

### 2.5 Evans blue extravasation

Evans blue (EB, a marker for blood–brain barrier disruption) extravasation is a hallmark of blood–brain barrier disruption. Evans blue extravasation was measured similarly as our previous study (16). Briefly, EB (2%, 5 mL/kg) was injected into the left femoral vein under anesthesia and allowed to circulate for 60 min. Then the mice were anesthetized and transcardially perfused with phosphate-buffered saline (PBS). Brain tissues were separated and weighted, then homogenized in PBS and centrifuged at 15,000 g for 30 min. The supernatant was mixed with equal volume of trichloroacetic acid, then incubated overnight at 4 °C and centrifuged again at 15,000 g for 30 min. The supernatant was collected and measured by SpectraMax M5 microplate reader (Molecular Devices, USA) at 620 nm.

### 2.6 Brain water content

A common wet/dry method was used to evaluate brain edema as our previous studies (14, 15). Briefly, 3 days after surgery, mice were deep anaesthetized. Brains were harvested and quickly divided into three parts including ipsilateral and contralateral hemisphere, and cerebellum. Each sample was weighted immediately to get wet weight and then dried in an oven at 100 °C for 24 h to obtain dry weight. The brain water content was calculated as (wet weight–dry weight)/wet weight×100%.

### 2.7 Determination of malondialdehyde (MDA), glutathione peroxidase (GPx) and superoxide dismutase (SOD)

The brain samples were homogenized in 2 mL PBS and centrifuged at 12,000 g for 20 min at 4 °C. The supernatant was collected. Protein concentrations were measured using a BCA kit (Pierce, USA). The MDA content, GPx and SOD activities were measured spectrophotometrically with commercial kits according the manufacture’s protocols (Beyotime, China). The content of MDA was expressed as nmol/mg protein and the activities of GPx and SOD were showed as U/mg protein, respectively.

### 2.8 Western blot analysis

Western blot assay was carried out similarly as our previous report (14). First, brain tissues were homogenized in 0.5 mL ice-cold RIPA buffer and the supernatants were collected through centrifugation at 12,000 g for 20 min at 4 °C. For the detection of Nrf2 protein, nuclear and cytoplasmic proteins were extracted following the protocol of the nuclear and cytoplasmic protein extraction kit (Beyotime, China). Protein concentrations were determined using BCA method. Then, proteins were boiled in denaturing sample buffer. Equal amounts of protein were separated by 12% SDS–polyacrylamide gel electrophoresis and then transferred to nitrocellulose membranes.

After blocking with 5% skim milk in TBST for 2 h, the membranes were incubated in blocking buffer overnight at 4 °C with following primary antibodies: Nrf2 (1:1000; Abcam), HO-1 (1:2000; Abcam), NQO-1 (1:1000; Abcam), Histone 3 (1:1000; Cell Signaling Technology, USA), and *β*-actin (1:5000; BioworldTechnology, USA). After being washed three times with TBST, the membranes were further incubated with horseradish peroxidase-conjugated secondary antibody at room temperature for 2 h. Immunoblots were visualized with ECL Plus reagent kit (Beyotime, China). Densities of the bands were quantified with ImageJ software.

### 2.9 Iba-1 immunohistochemistry

Iba-1 immunohistochemistry was carried out as our previous study (15). Brain sections were mounted on gelatin-coated slides and washed three times with PBS for 10 min each. According to the immunostaining protocol, slides were incubated in 10% normal goat serum blocking buffer for 2 h. They were incubated in a solution containing rabbit anti-mouse Iba-1 antibody (1:200, Wako, Japan) at 4 °C overnight. Slides were washed with PBS three times for 10 min and then incubated in a solution containing secondary antibody Alexa Flour 594 (1:200; Invitrogen, USA) for 1 h at room temperature. Slides were washed three times with PBS and coverslipped. Two pathologists blinded to the experimental groups visualized and counted the Iba-1 positive cells in the brain sections under a fluorescence microscope (Eclipse Ti, Nikon Corporation, Tokyo, Japan). The data were presented as Iba-1 positive cells/(200 magnification visual field).

### 2.10 Enzyme-linked immunosorbent assay (ELISA)

The brain tissues were harvested and mechanically homogenized in 1 mL lysate buffer, and then centrifuged at 12,000 g for 20 min at 4 °C. The supernatant was collected for further analysis. The total protein was determined using bicinchoninic acid assay kit (Pierce, USA). The levels of inflammatory cytokines were determined using specific enzyme-linked immunosorbent assay kits (TNF-α and IL-1β, Westang, Shanghai, China) for mouse according to the manufacturer instructions. Results were expressed as picogram per milligram protein.

### 2.11 Statistical analysis

All results are presented as mean ± standard error of the mean (SEM). Exact sample size (*n* = 6 per group) is stated for all quantitative analyses. For intergroup comparisons, one-way or two-way analysis of variance (ANOVA) was applied. Prior to ANOVA, normality of data distribution and homogeneity of variance were verified; all datasets met the assumptions for ANOVA analysis. Bonferroni’s multiple comparison test was used as the *post-hoc* test to identify differences between individual groups. All statistical analyses were performed using GraphPad Prism 5. A two-tailed *p*-value < 0.05 was considered statistically significant.

## 3 Results

### 3.1 Astaxanthin improved neurobehavioral function and suppressed neural cell degeneration after ICH

The experimental procedures of our study were presented as Figure 1A. Of note, no significant difference in primary hematoma volume was observed among the three groups at each detected time point (data not shown), indicating that the neuroprotective effects of ATX were mainly attributed to the reduction of secondary brain injury rather than altered primary hemorrhagic damage. To investigate the neuroprotective role of astaxanthin in ICH mice, ATX (100 mg/kg) was administrated through intraperitoneal injection to mice at 30 min after ICH induction. Neurobehavioral function was tested in each group at 0, 1d, 3d and 7d post-surgery using a 24-point scoring system. No difference in baseline neurobehavioral function was seen in any groups before surgery (Figure 1B). As shown in Figure 1B, neurological deficits were severe at 1d, 3d and 7d after ICH induction. However, ATX treatment significantly reduced neurological deficits at all assess time points after ICH when compared with vehicle-treated ICH group (Figure 1B).

To determine whether ATX had a protective role on histopathology level, Fluoro-Jade C (FJC) staining was employed to examine the neural cells degeneration in brain tissue at 3 days after ICH. As shown in Figures 1C,D, few FJC positive cells (green) were seen in the sham group mice, while FJC positive cells were greatly increased in perihematomal regions after ICH. However, ATX treatment remarkably decreased neural cell degeneration after ICH. Therefore, these results indicated ATX exerted neuroprotection through inhibiting neural cell degeneration.

### 3.2 Astaxanthin reduced blood–brain barrier disruption and brain edema after ICH

Destruction of the blood–brain barrier (BBB) and brain edema were considered as crucial pathological processes contributing to ICH-induced brain injury. The BBB disruption was assessed by Evans blue dye extravasation at 24 h after ICH. Figure 1E showed that there was little Evans blue dye leakage into brain tissue in the sham group. Our results further showed that Evans blue dye leakage into brain tissue was greatly increased after ICH induction, which was dramatically ameliorated by ATX administration (Figure 1E).

Brain water content was often used to determine the severity of brain edema. As a result of BBB disruption, brain water content in ipslateral hemisphere brain tissue was significantly increased at 3 days after ICH induction when compared with sham group. ATX treatment greatly reduced brain water content in ipslateral hemisphere brain tissue (Figure 1F).

### 3.3 Astaxanthin treatment decreased microglia activation and pro-inflammatory mediators after ICH

**Figure 2:**
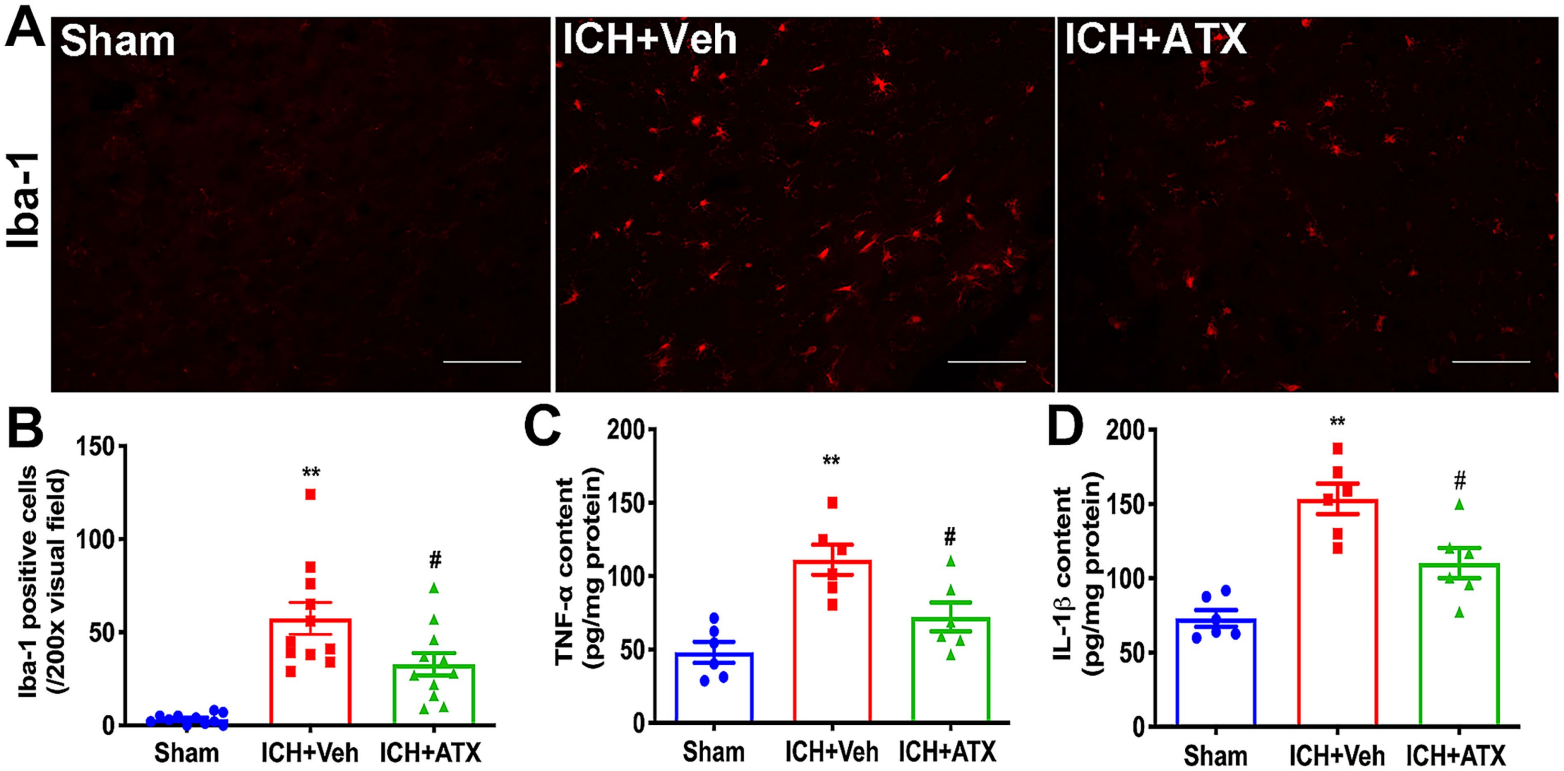
Astaxanthin (ATX) treatment decreased microglia activation and pro-inflammatory mediators after ICH. **(A)** Representative images of Iba-1 staining showed the activated microglia in the experimental groups. Scale bar, 50 μm. **(B)** Quantification of Iba-1 positive cells showed that treatment with ATX significantly reduced microglia activation at 3 days after ICH. **(C)** ATX treatment significantly reduced the concentration of TNF-α at 3 days after ICH. **(D)** ATX treatment greatly decreased the concentration of IL-1β at 3 days after ICH. Data were presented as mean ± SEM. Individual data points of each mouse are overlaid on the plots to reflect data variability and sample distribution. *n* = 6 mice/group. **p < 0.01 versus Sham group; ^#^*p* < 0.05, versus ICH + Veh group.

To investigate whether ATX has a neuroprotective effect on inhibiting neuroinflammation, we further studied microglia activation and the expression of pro-inflammatory mediators at 3 days after ICH. Results showed that microglia were significantly activated after ICH and were dramatically reduced by ATX treatment at 3 days after ICH (Figures 2A,B). Furthermore, ATX treatment significantly reduced the production of pro-inflammatory mediators TNF-α (Figure 2C) and IL-1β (Figure 2D) at 3 days after ICH. These results indicated that ATX reduced neuroinflammation after ICH.

### 3.4 Astaxanthin attenuated oxidative stress caused by ICH

**Figure 3:**
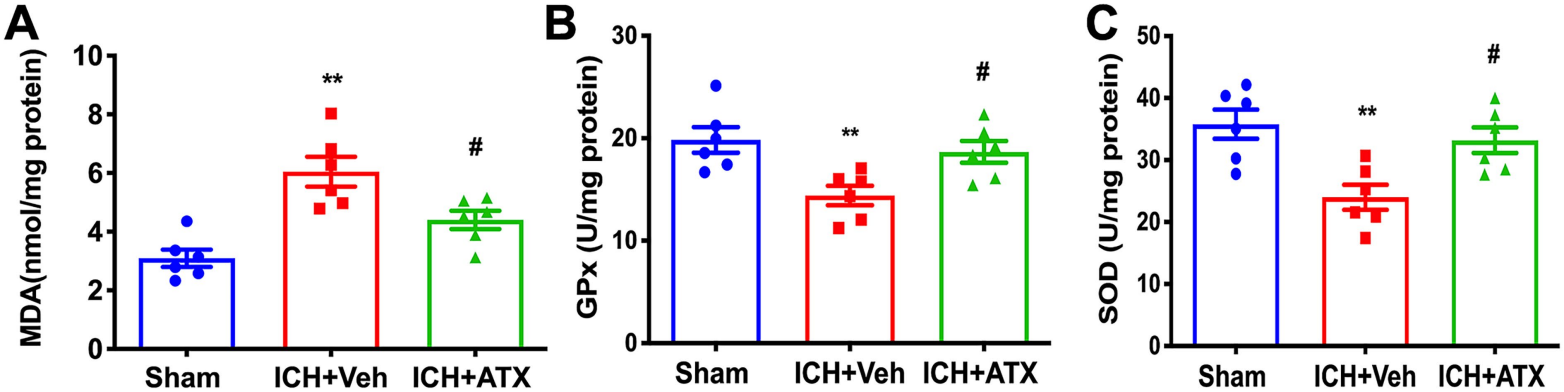
Treatment of astaxanthin (ATX) attenuated oxidative stress caused by ICH. Oxidative stress was assessed at 24 h after ICH by the level of malondialdehyde (MDA), the activity of glutathione peroxidase (GPx) and superoxide dismutase (SOD) in the present study. **(A)** The level of MDA was significantly inhibited by ATX treatment after ICH. **(B)** ATX treatment remarkably restored the activity of GPx after ICH. **(C)** The activity of SOD was greatly increased by ATX administration after ICH. Data were expressed as mean ± SEM. Individual data points of each mouse are overlaid on the plots to reflect data variability and sample distribution. *n* = 6 mice/group. **p < 0.01 versus Sham group; ^#^*p* < 0.05, versus ICH + Veh group.

To determine the effects of ATX on oxidative stress, we measured the level of MDA, the activities of GPx and SOD at 24 h after ICH in the present study. As shown in Figure 3, ICH remarkably increased the levels of MDA (Figure 3A), and greatly inhibited the activities of GPx (Figure 3B) and SOD (Figure 3C) when compared with sham group. However, treatment with ATX significantly reduced the level of MDA (Figure 3A), and remarkably restored the activities of GPx (Figure 3B) and SOD (Figure 3C) when compared with ICH + Veh group.

### 3.5 Astaxanthin promoted Nrf2 protein translocation from cytoplasm to nucleus and increased the expression of Nrf2 downstream factors after ICH

**Figure 4:**
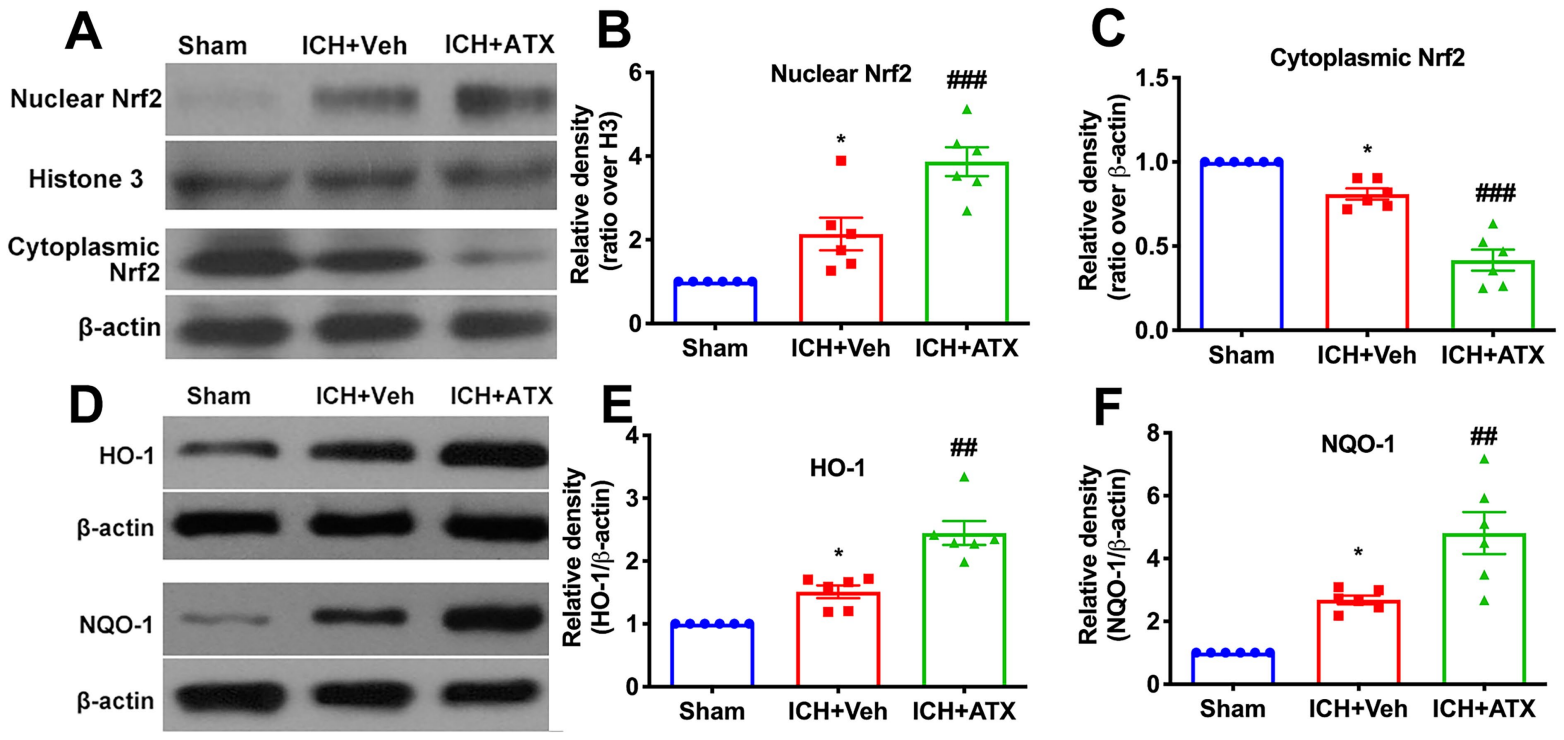
Astaxanthin (ATX) promoted translocation of Nrf2 from cytoplasm to nucleus and increased the expressions of Nrf2 downstream factors. **(A)** Representative western blot assay showed the level of nuclear and cytoplasmic Nrf2 protein in the experimental groups. Histone 3 (H3) and *β*-actin were used as loading control. **(B)** Quantitative analysis revealed that Nrf2 protein in nucleus was increased after ICH and further increased after ATX treatment. **(C)** Quantitative analysis showed that Nrf2 protein in cytoplasm was decreased after ICH and further decreased after ATX treatment. **(D)** Representative western blot showed the expressions of HO-1 and NQO-1 protein in experimental groups. *β*-actin was used as loading control. **(E)** Quantitative analysis revealed that HO-1 protein was upregulated after ICH and further greatly increased after ATX treatment. **(F)** Quantitative analysis revealed that NQO-1 protein was upregulated after ICH and further significantly increased after ATX treatment. Data were expressed as mean ± SEM. Individual data points of each mouse are overlaid on the plots to reflect data variability and sample distribution. *n* = 6 mice/group. *p < 0.05 versus Sham group; ^##^*p* < 0.01 and ^###^*p* < 0.001, versus ICH + Veh group.

It has been reported that Nrf2 plays an important role in the regulation of oxidative stress (12). Our above-mentioned results indicated ATX inhibited oxidative stress induced by ICH. Nrf2 has been proven to exert neuroprotection in ICH (10). So, we further investigated whether ATX provided neuroprotection involving Nrf2 pathway in ICH. Our results showed ATX treatment significantly increased Nrf2 protein in nucleus (Figures 4A,B) and dramatically decreased Nrf2 protein in cytoplasm (Figures 4A,C) after ICH, which may indicated that ATX promoted Nrf2 nuclear translocation from cytoplasm.

To explore whether ATX could further affect Nrf2 downstream factors, we investigated the effect of ATX on expression of Nrf2 downstream proteins such as HO-1 and NQO-1. Western blot analysis revealed that both HO-1 and NQO-1 proteins were increased after ICH, and further greatly upregulated after ATX treatment in ICH mice (Figures 4D–F). These results demonstrated that ATX treatment was associated with Nrf2 nuclear translocation and upregulation of its downstream antioxidant proteins HO-1 and NQO-1 after ICH, suggesting a close association between the Nrf2-ARE cascade and the protective effects of ATX.

## 4 Discussion

In the present study, we proved the neuroprotection of astaxanthin in ICH mice. Our results showed that ATX treatment after ICH significantly reduced neurological deficits, neural cell degeneration, blood–brain barrier disruption and brain edema. We further demonstrated that ATX exerted anti-inflammatory effects by inhibiting microglia activation and production of pro-inflammatory mediators. Moreover, ATX administration attenuated ICH-induced oxidative stress potential by activating Nrf2 signaling pathway and further stimulating antioxidant enzymes, including HO-1, NQO-1, GPx and SOD. To the best of our knowledge, these results demonstrated for the first time that astaxanthin ameliorated ICH-induced brain injury in mice potential by activating Nrf2-ARE pathway.

ICH causes primary mechanical injury and secondary brain injury that occurs immediately after primary damage. There are several parallel pathophysiologic processes responsible for secondary brain injury after ICH, including blood–brain barrier disruption, brain edema formation, oxidative stress, microglia activation, inflammatory responses and cell death (1, 2, 14). These pathologic events finally caused neurological dysfunction in ICH patients. Therapeutic strategies that targeted at these pathophysiologic processes could be very effective in reducing brain injury after ICH.

Astaxanthin, known as a dietary carotenoid and strong antioxidant, is widely distributed in crustaceans, algae, shellfish and some plants. Food and Drug Administration of USA have approved ATX as a feed additive and dietary supplement for its treatment effect and safety usage in human (17). ATX has been reported to have multiple pharmacological effects, including anti-oxidative stress, anti-inflammation, and anti-cell death (3, 5). ATX has been shown to exert neuroprotection in several CNS diseases, including TBI, ischemic stroke and SAH (3–5). However, no study has been carried out to explore the efficacy of ATX on ICH-induced brain injury. In the present study, we investigated the protective effects of ATX through collagenase-induced ICH model. ATX was administrated in a dose of 100 mg/kg through intraperitoneal injection at 30 min after ICH induction. The dose and route of ATX were selected according to previous studies (3, 5). We found that ATX treatment significantly reduced neurological deficits, neural cells degeneration, blood–brain barrier disruption and brain edema after ICH (Figure 1). Neuroinflammation played important role in the pathology and progression of ICH, with activation of microglia and production of inflammatory mediators (10, 18). Inflammatory mediators such as TNF-α and IL-1β exacerbated brain injury after ICH (14). So we further explored the effect of ATX on neuroinflammation post ICH. As expected, results showed that ATX treatment attenuated microglial activation (Figures 2A,B) and inhibited the productions of TNF-α and IL-1β (Figures 2C,D) at 3 days after ICH.

Oxidative stress caused by reactive oxygen species (ROS) played a prominent role in secondary brain injury post ICH (1, 2, 10). MDA represented the major product of lipid peroxidation in oxidative stress. MDA is widely used as an index of lipid peroxidation. GPx and SOD are well-known intracellular antioxidant enzymes with the capacity to scavenge free radicals and ROS (12). We explored whether ATX attenuated oxidative stress in ICH brain through its anti-oxidative property. Our results showed that ICH increased the MDA levels and inhibited the activities of GPx and SOD. ATX treatment significantly inhibited the level of MDA and increased the activities of GPx and SOD after ICH (Figure 3). The above findings indicated that astaxanthin played important roles in inhibition of inflammation, oxidative stress and cell death after ICH.

The transcription factor Nrf2 is a master regulator of antioxidative and detoxification processes (12). In stress condition, Nrf2 is activated and translocates from cytoplasm into nucleus. Then through binding to ARE, Nrf2 induces the expression of many detoxification and antioxidant enzymes, including HO-1 and NQO-1, SOD and GPx (10, 12). Nrf2-ARE pathway, as a key player in antioxidative homeostasis, has been proven to be activated and exert neuroprotection in ICH (10). Nrf2 absence exacerbated brain injury in ICH mice (19). Moreover, a recent study proved that ATX inhibited early brain injury through activating Nrf2-ARE pathway after SAH in rats (13). So we speculated that ATX might exert neuroprotective properties by activating Nrf2-ARE pathway after ICH. We investigated the changes of Nrf2-ARE pathway after ATX treatment. In agreement with this assumption, our results showed that ATX significantly enhanced translocation of Nrf2 from cytoplasm to nuclear (Figures 4A–C). In addition, we found the expression of Nrf2 downstream antioxidant enzymes (HO-1 and NQO-1) were markedly increased after ICH and further greatly upregulated after ATX treatment (Figures 4D–F). Our data revealed a strong correlation between the altered activity of Nrf2-ARE pathway and the neuroprotective phenotypes of ATX. However, since Nrf2 inhibitor or gene knockout models were not applied in the present study, we cannot fully confirm the causal relationship between Nrf2-ARE activation and ATX-mediated neuroprotection. Further work is needed to investigate whether Nrf2-ARE pathway or other signaling pathways are involved in the protective mechanisms of ATX in ICH by specific inhibitors or gene knockout models. Further studies should focus on determining the optimal concentration and dose of ATX for maximal protection in ICH.

Limitations of the present study should be acknowledged. First, we only adopted a single dose (100 mg/kg) and a single administration time point (30 min after ICH) of astaxanthin in this work. The dose–response relationship and therapeutic time window of ATX for ICH were not systematically explored. Therefore, the optimal dosage, administration frequency and time window for clinical translation remain unclear, and further dose-gradient and time-gradient experiments are required in future studies. Second, the neurological function assessment in our study was only performed within 7 days after ICH. Long-term functional recovery (over 7 days) was not evaluated. Given that long-term neurological rehabilitation is a key indicator for evaluating the prognosis of ICH, the short follow-up period is a major limitation of this study. Extended the follow-up time is needed in future animal experiments. Third, only male C57BL/6 mice were used in all animal experiments. Numerous studies have confirmed that sex is an important confounding factor in the occurrence, progression and pathological manifestations of ICH. The lack of female mouse groups reduces the generalizability of our findings. Subsequent studies need to include both male and female animals to verify the reproducibility of the current results.

In summary, the present study demonstrated that systemic administration of ATX reduced neurological deficits, neural cell degeneration, blood–brain barrier disruption, brain edema, neuroinflammation and oxidative stress after ICH. Our results further indicated that Nrf2 pathway activation was potentially involved in the neuroprotective effects of ATX treatment after ICH. These results also suggested ATX might be an effective therapeutic drug for the treatment of ICH by the potential mechanism of activating Nrf2-ARE pathway and deserved further investigation.

## Data Availability

The original contributions presented in the study are included in the article/supplementary material, further inquiries can be directed to the corresponding author.
